# An efficient automated parameter tuning framework for spiking neural networks

**DOI:** 10.3389/fnins.2014.00010

**Published:** 2014-02-04

**Authors:** Kristofor D. Carlson, Jayram Moorkanikara Nageswaran, Nikil Dutt, Jeffrey L. Krichmar

**Affiliations:** ^1^Department of Cognitive Sciences, University of California IrvineIrvine, CA, USA; ^2^Brain CorporationSan Diego, CA, USA; ^3^Department of Computer Science, University of California IrvineIrvine, CA, USA

**Keywords:** spiking neural networks, parameter tuning, evolutionary algorithms, GPU programming, self-organizing receptive fields, STDP

## Abstract

As the desire for biologically realistic spiking neural networks (SNNs) increases, tuning the enormous number of open parameters in these models becomes a difficult challenge. SNNs have been used to successfully model complex neural circuits that explore various neural phenomena such as neural plasticity, vision systems, auditory systems, neural oscillations, and many other important topics of neural function. Additionally, SNNs are particularly well-adapted to run on neuromorphic hardware that will support biological brain-scale architectures. Although the inclusion of realistic plasticity equations, neural dynamics, and recurrent topologies has increased the descriptive power of SNNs, it has also made the task of tuning these biologically realistic SNNs difficult. To meet this challenge, we present an automated parameter tuning framework capable of tuning SNNs quickly and efficiently using evolutionary algorithms (EA) and inexpensive, readily accessible graphics processing units (GPUs). A sample SNN with 4104 neurons was tuned to give V1 simple cell-like tuning curve responses and produce self-organizing receptive fields (SORFs) when presented with a random sequence of counterphase sinusoidal grating stimuli. A performance analysis comparing the GPU-accelerated implementation to a single-threaded central processing unit (CPU) implementation was carried out and showed a speedup of 65× of the GPU implementation over the CPU implementation, or 0.35 h per generation for GPU vs. 23.5 h per generation for CPU. Additionally, the parameter value solutions found in the tuned SNN were studied and found to be stable and repeatable. The automated parameter tuning framework presented here will be of use to both the computational neuroscience and neuromorphic engineering communities, making the process of constructing and tuning large-scale SNNs much quicker and easier.

## Introduction

Although much progress has been made in simulating large-scale spiking neural networks (SNNs), there are still many challenges to overcome before these neurobiologically inspired algorithms can be used in practical applications that can be deployed on neuromorphic hardware (Boahen, 2005; Markram, 2006; Nageswaran et al., 2010; Indiveri et al., 2011). Moreover, it has been difficult to construct SNNs large enough to describe the complex functionality and dynamics found in real nervous systems (Izhikevich and Edelman, 2008; Krichmar et al., 2011; Eliasmith et al., 2012). Foremost among these challenges are the tuning and stabilization of large-scale dynamical systems, which are characterized by many state values and open parameters (Djurfeldt et al., 2008). The task of tuning SNNs is becoming more difficult as neuroscientists move away from simpler models toward more realistic, but complex models to describe the properties of network elements (van Geit et al., 2008). For example, many modelers have moved away from simple “integrate and fire” neuron models to models which capture a wider range of neuronal dynamics, but have more open parameters (Izhikevich, 2003; Brette and Gerstner, 2005). A similar shift in complexity is occurring when simulating synaptic plasticity (Abbott and Nelson, 2000), as new types of plasticity models such as homeostatic synaptic scaling (Watt and Desai, 2010; Carlson et al., 2013), short-term plasticity (Mongillo et al., 2008), and spike-timing dependent plasticity (STDP) (Song et al., 2000; van Rossum et al., 2000) are being incorporated into SNNs. In addition, network topologies are shifting from conventional feed-forward connectivity to recurrent connectivity, which have more complex dynamics and require precise tuning of synaptic feedback for stable activity (Seung et al., 2000).

For these reasons, the process of hand-tuning SNNs is often extremely time-consuming and inefficient which has led to interest among researchers in automating this process. To address these challenges, we present an automated tuning framework that utilizes the parallel nature of graphics processing units (GPUs) and the optimization capabilities of evolutionary algorithms (EAs) to tune open parameters of SNNs in a fast and efficient manner.

The present article describes a means to automate parameter tuning of spiking neural networks which are compatible with present and future neuromorphic hardware. However, it is important to first examine the role SNN models play in the development of neuromorphic hardware. Recent neuromorphic science funding initiatives, such as the SyNAPSE project in the USA and the FACETS/BrainScaleS projects in Europe, have resulted in the construction of neuromorphic chips. Not surprisingly, the research groups involved in producing these neuromorphic hardware devices have also spent a great deal of time building software simulation and interface frameworks (Amir et al., 2013; Thibeault and Srinivasa, 2013). This is because the novel hardware requires new software environments and methodologies to run applications (Brüderle et al., 2011). There are two main software development tasks required to run neuromorphic applications on a hardware device. First, the neuromorphic application must be designed and tuned to perform a particular cognitive or computational function. This is the focus of our present study. Second, the model description of the neuromorphic application must be mapped onto the neuromorphic hardware device computing elements. There have been a number of recent studies that have applied various optimization techniques to solve this mapping problem with some success (Ehrlich et al., 2010; Sheik et al., 2011; Gao et al., 2012; Neftci et al., 2013). Although both tasks are integral to developing neuromorphic hardware applications, the latter is outside the scope of present study.

There has been a great deal of work in the computational neuroscience community on automating the process of parameter tuning neuronal models. A variety of different methodologies have been used to automate parameter tuning in neural models, many of which are summarized in the review by van Geit et al. (2008). Svensson et al. (2012) fit a nine-parameter model of a filter-based visual neuron to experimental data using both gradient following (GF) methods and EAs. Some groups have used optimization techniques to tune ion channels kinetics for compartmental neurons (Hendrickson et al., 2011; Ben-Shalom et al., 2012) while other groups have used quantum optimization techniques and EAs to tune more abstract networks of neurons (Schliebs et al., 2009, 2010). Additionally, brute force methods of searching the parameter space were used to examine a three-neuron model of a lobster stomatogastric circuit by creating large databases of compartmental neurons with varying membrane conductance values and testing the resulting functional behavior of this circuit (Prinz et al., 2003, 2004). Some automated parameter-search tools have been developed as interfaces between neural simulators and the optimization methods used to tune them such as Neurofitter (van Geit et al., 2008). Other tools allow for the automatic compilation of very large sets of simulation runs across a wide range of parameter values and experimental conditions (Calin-Jageman and Katz, 2006).

Unlike these parameter tuning methodologies, which have been applied to small neural circuits, single neurons or networks of hundreds of neurons, our focus is the automated tuning of much *larger* neural systems (on the scale of 10^3^–10^6^ neurons). Neural networks at these scales become more useful for the description of cognitive models and closer to the scale of SNNs currently being designed to run on neuromorphic hardware (Esser et al., 2013; Thibeault and Srinivasa, 2013). Recent work by Rossant et al. (2011) and Eliasmith et al. (2012) has focused on tuning large-scale SNNs; we compare these approaches with our tuning framework in the discussion section.

A parallel line of research in automated parameter tuning has taken place where larger, more abstract artificial neural networks (ANNs) are constructed using EAs (Fogel et al., 1990). The building of ANNs using EAs can be broken into two basic methodologies: direct encoding and indirect encoding. Much work has been done using the direct encoding approach, where the genetic description of the individual, or the genotype, is directly mapped to the actual traits of the individual, or the phenotype (Hancock, 1992; Gomez and Miikkulainen, 1997; Stanley and Miikkulainen, 2002). An EA is said to use direct encoding when there is a one-to-one correspondence between parameter values, like synaptic weight values and genotype values. Drawbacks of this approach include poor genotype scaling for large network encodings and very large parameter spaces due to the lack of geometrical constraints of the networks. Alternatively, indirect encoding allows the genotype to specify a rule or method for growing the ANN instead of specifying the parameter values directly (Husbands et al., 1998; Beer, 2000; Floreano and Urzelai, 2001; Stanley and Miikkulainen, 2003). NeuroEvolution of Augmented Topologies (NEAT) and HyperNEAT use indirect encoding to evolve network topologies, beginning with a small network and adding complexity to that network as evolution progresses (Stanley and Miikkulainen, 2002; Stanley et al., 2009; Clune et al., 2011; Risi and Stanley, 2012). HyperNEAT has been used to encode networks with as many as 8 million connections and networks evolved using NEAT have been used in food-gathering tasks (Stanley et al., 2009), in a checkers-playing ANN that exhibits topographic mappings (Gauci and Stanley, 2010), and in evolving robot gaits in hardware (Yosinski et al., 2011). The present study utilizes the indirect encoding approach, in which the learning parameters are evolved, as opposed to the direct encoding approach where the synaptic weights are evolved. This allows for a large reduction in the parameter space. Although EAs are an effective tool for constructing ANNs, they often require long execution times to produce well-tuned networks. A number of parallel computing techniques can be used to reduce the execution time of EAs, however, this paper focuses mainly on parallelization via GPU computing.

With the development of mature GPU parallel computing platforms like CUDA (Nickolls et al., 2008) and OpenCL (Stone et al., 2010), GPU accelerated algorithms have been applied to a variety of tasks in scientific computing. GPU acceleration has been used to increase the throughput of EAs (Maitre et al., 2009), simulate neural field models of the primary visual cortex V1 (Baladron et al., 2012), and search parameter spaces in bio-inspired object-recognition models (Pinto et al., 2009). In addition to these applications, a number of research groups in the computational neuroscience community (Brette and Goodman, 2012) have developed and implemented parallel implementations of SNNs on GPUs (Bernhard and Keriven, 2006; Fidjeland et al., 2009; Nageswaran et al., 2009b; Bhuiyan et al., 2010; Han and Taha, 2010; Hoffmann et al., 2010; Yudanov et al., 2010; Ahmadi and Soleimani, 2011; Nowotny, 2011; Thibeault et al., 2011; de Ladurantaye et al., 2012; Mirsu et al., 2012; Pallipuram et al., 2012). GPU-driven SNN simulators have already been used in SNN models of the basal forebrain (Avery et al., 2012), the basal ganglia (Igarashi et al., 2011), the cerebellum (Yamazaki and Igarashi, 2013), and the olfactory system (Nowotny, 2010).

Our present study drastically decreases the time it takes to tune SNN models by combining a freely available EA library with our previous work (Nageswaran et al., 2009b; Richert et al., 2011), which consisted of a parallelized GPU implementation of an SNN simulator. Although other research groups have used EAs and GPUs to tune SNNs (Rossant et al., 2011), our approach is more general as it tunes a variety of SNN parameters and utilizes fitness functions that can be broadly applied to the behavior of the entire SNN. As a proof of concept, we introduce a parameter tuning framework to evolve SNNs capable of producing self-organized receptive fields similar to those found in V1 simple cells in response to patterned inputs. An indirect encoding approach was utilized as the parameters tuned in the SNN governed Hebbian learning, homeostasis, maximum input stimulus firing rates, and synaptic weight ranges. A performance analysis compared the parallelized GPU implementation of the tuning framework with the equivalent central processing unit (CPU) implementation and found a speedup of 65× (i.e., 0.35 h per generation vs. 23.5 h per generation) when SNNs were run concurrently on the GPU. Using affordable, widely-accessible GPU-powered video cards, the software package presented here is capable of tuning complex SNNs in a fast and efficient manner. The automated parameter tuning framework is publicly available and could be very useful for the implementation of large-scale SNNs on neuromorphic hardware or for the development of large-scale SNN simulations that describe complex brain functions.

## Methods

### GPU accelerated SNNs in CARLsim

**Figure 1:**
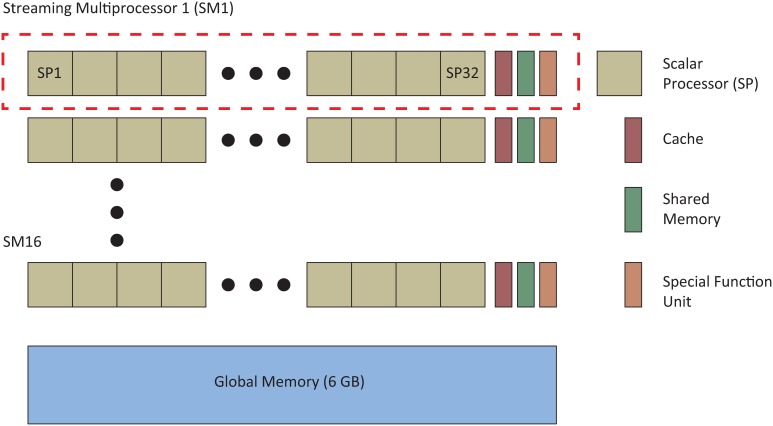
**A simplified diagram of NVIDIA CUDA GPU architecture (adapted from Nageswaran et al., 2009a,b)**. Our simulations used an NVIDIA Tesla 2090 GPU that had 16 streaming multiprocessors (SM) made up of 32 scalar processors (SPs) and 6 GB of global memory.

An important feature of the automated parameter tuning framework is the ability to run multiple SNNs in parallel on the GPU, allowing significant acceleration of the EA evaluation phase. We first briefly review the approaches CARLsim uses to run SNNs in parallel before describing the general layout of the automated parameter tuning framework and describe how a researcher would use the tool to tune SNNs. Figure 1 shows the basic CUDA GPU architecture, which consists of a multiple streaming multiprocessors (SMs) and a global memory, accessible to all SMs. Each SM is comprised of multiple floating-point scalar processors (SPs), at least one special function unit (SFU), and a cache/shared memory. CUDA code is distributed and executed in groups of 32 threads called warps. Each SM has at least one warp scheduler that ensures maximum thread concurrency by switching from slower to faster executing warps. Our simulations utilized an NVIDIA Tesla M2090 GPU with 6 GB of global memory, 512 cores (each operating at 1.30 GHz) grouped into 16 SMs (32 SPs per SM), and a single precision compute power of 1331.2 GFLOPS.

The CARLsim parallel GPU implementation was written to optimize four main performance metrics: parallelism, memory bandwidth, memory usage, and thread divergence which are discussed in greater detail in (Nageswaran et al., 2009a). The term parallelism refers to both the degree to which the application data is mapped to parallel threads and the structure of the mapping itself. CARLsim utilizes both neuronal parallelism (N-parallelism), where individual neurons are mapped to processing elements and simulated in parallel, and synaptic parallelism (S-parallelism), where synaptic data are mapped to processing elements and simulated in parallel. Anytime a neuronal state variable is updated, N-parallelism is used, and anytime a weight update is necessary, S-parallelism is used. Sparse representation techniques such as the storage of SNN data structures using the reduced Address Event Representation (AER) format and the use of a circular queue to represent firing event data decrease both memory and memory bandwidth usage. GPUs execute many threads concurrently (1536 threads per SM in the Tesla M2090) and manage these threads by providing a thread scheduler for each SM which organizes groups of threads into warps. Thread/warp divergence occurs when threads in a single warp execute different operations, forcing the faster executing threads to wait until the slower threads have completed. In CARLsim, thread/warp divergence is minimized during diverging loop executions by buffering the data until all threads can execute the diverging loop simultaneously.

### Automated parameter tuning framework description

**Figure 2:**
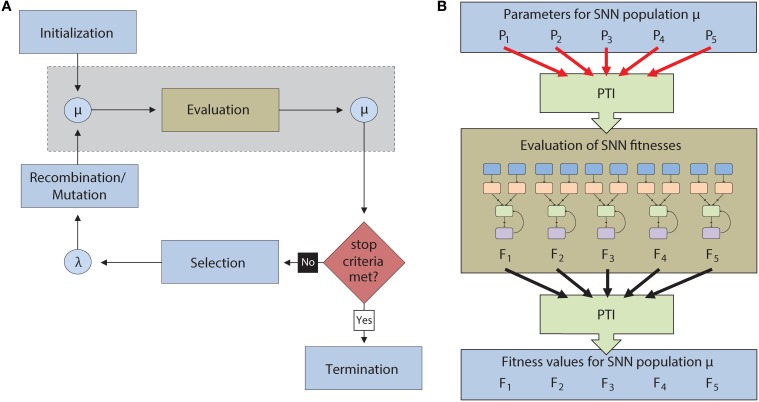
**(A)** Flow chart for the execution of an Evolutionary Algorithm (EA). A population of individuals (μ) is first initialized and then evaluated. After evaluation, the most successful individuals are selected to reproduce via recombination and mutation to create an offspring generation (λ). The offspring then become parents for a new generation of the EA. This continues until a termination condition is reached. The light blue boxes denote operations that are carried out serially on the CPU while the light brown box denotes operations carried out in parallel on the GPU. The operations inside the dotted gray box are described in greater detail in **(B)**. **(B)** Description of the automated parameter tuning framework consists of the CARLsim SNN simulator (light brown), the EO computational framework (light blue), and the Parameter Tuning Interface (PTI) (light green). The PTI passes tuning parameters (*P_N_*) to CARLsim for evaluation in parallel on the GPU. After evaluation, fitness values (*F_N_*) are passed from CARLsim back to EO via the PTI.

To test the feasibility of an automated parameter tuning framework, our group used EAs to tune open parameters in SNNs running concurrently on a GPU. As a proof of concept, the SNNs were evolved to produce orientation-dependent stimulus responses similar to those found in simple cells of the primary visual cortex (V1) through the formation self-organizing receptive fields (SORFs). The general evolutionary approach was as follows: (1) A population of neural networks was created, each with a unique set of neural parameter values that defined overall behavior. (2) Each SNN was then ranked based on a fitness value assigned by the objective function. (3) The highest ranked individuals were selected, recombined, and mutated to form the offspring for the next generation. (4) This process continued until a desired fitness was reached or until other termination conditions were met (Figure 2A).

The automated parameter tuning framework consisted of three software packages and is shown in Figure 2B. The framework includes: (1) the CARLsim SNN simulator (Richert et al., 2011), (2) the Evolving Objects (EO) computational framework, a publically available evolutionary computation toolkit (Keijzer et al., 2002), and (3) a Parameter Tuning Interface (PTI), developed by our group, to provide an interface between CARLsim and EO. Evolving Objects is available at http://eodev.sourceforge.net/ and both CARLsim and the PTI are available at http://www.socsci.uci.edu/~jkrichma/CARLsim/. The EO computational framework runs the evolutionary algorithm on the user-designated parameters of SNNs in CARLsim. The PTI allows the objective function to be calculated independent of the EO computation framework. Parameter values are passed from the EO computation framework through the PTI to the SNN in CARLsim where the objective function is calculated. After the objective function is executed, the results are passed from the SNN in CARLsim through the PTI back to the EO computation framework for processing by the EA. With this approach, the fitness function calculation, which involves running each SNN in the population, can be run in parallel on the GPU while the remainder of EA calculations can be performed using the CPU (Figure 2B).

### Using the parameter tuning interface

In addition to providing a means for CARLsim and EO to exchange data, the PTI hides the low level description and configuration of EO from the user by providing a simple application programming interface (API). Before using the PTI, the user must have a properly configured EO parameter file, which is a plain text file that provides the user with control over an EO configuration. An example of an EO parameter file is shown in Supplementary 1 of the supplementary materials. At execution, EO reads the parameter file to configure all aspects of the EA, including selection, recombination, mutation, population size, termination conditions, and many other EA properties. Beginners to EO can use the example EO parameter files included with the EO source code for the automated parameter tuning framework presented here. A sample program overview of the PTI and a summary of the PTI-API are included in Supplementary materials sections 2 and 3. Additional EO examples and documentation can be found online at http://eodev.sourceforge.net/eo/tutorial/html/eoTutorial.html. After creating a valid EO parameter file, the user is ready to use the PTI and CARLsim to tune SNNs.

### Evolving SNNs with V1 simple cell responses and SORF formation

**Figure 3:**
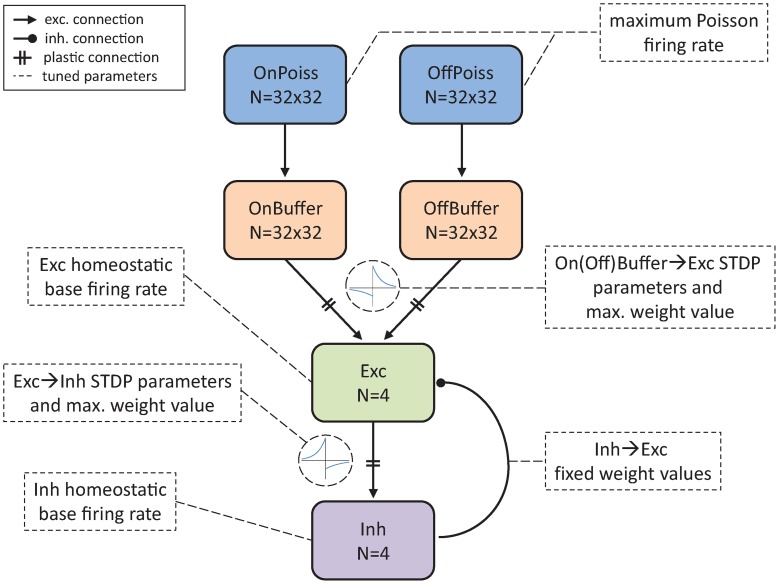
**Network architecture of the SNN tuned by the parameter tuning framework to produce V1 simple cell response and SORFs**. N represents the number of neurons used in different groups. E → E and E → I STDP curves are included to describe plastic On(Off)Buffer → Exc and Exc → Inh connections. Tuned parameters are indicated with dashed arrows and boxes.

As a proof of concept, the ability of the automated parameter tuning network to construct an SNN capable of producing SORFs and orientation-dependent stimulus responses was examined. This set of simulations was presented with grayscale counterphase gratings of varying orientations. The EO computation framework evolved SNN parameters that characterized spike-timing dependent plasticity (STDP), homeostasis, the maximum firing rates of the neurons encoding the stimuli, and the range of weight values for non-plastic connections. The network topology of the SNN, shown in Figure 3, modeled the visual pathway from the lateral geniculate nucleus (LGN) to the primary visual cortex (V1).

Each individual in the population participated in a training phase, where synaptic weights were modified according to STDP and homeostatic learning rules, and a testing phase where a multi-component objective function was used to evaluate an individual's ability to reproduce V1 simple-cell behavior. The training phase consisted of the presentation of 40 grayscale sinusoidal grating patterns of varying orientation (from π/40 to π) in random sequence to the SNN for approximately 100 min. Each pattern was presented to the network for 2 s while 1 Hz Poisson noise was applied to the network for 500 ms between pattern presentations. During the testing phase eight grating orientations (from π/8 to π) were presented to the network and the firing rate responses of the four output neurons in the Exc group were recorded. STDP and homeostasis were enabled during the training phase but were disabled for the testing phase. The evolutionary algorithm began with the random initialization of the parent population, consisting of 10 SNNs, and produced 10 offspring per generation. Ten SNN configurations ran in parallel. To evolve V1 simple cell responses, a real-valued optimization algorithm called Evolution Strategies (De Jong, 2002) was used with deterministic tournament selection, weak-elitism replacement, 40% Gaussian mutation and 50% crossover. Weak-elitism ensures the overall fitness monotonically increases each generation by replacing the worst fitness individual of the offspring population with the best fitness individual of the parent population. Fourteen parameters were evolved: four parameters associated with E → E STDP, four parameters associated with E → I STDP, the homeostatic target firing rates of the Exc and Inh groups, the strength of the fixed uniformly random On(Off)Buffer → Exc group connections, the strength of the plastic Exc → Inh group connections, the strength of the fixed uniformly random Inh → Exc group connections, and the maximum firing rate response to the input stimuli. The range of allowable values for each parameter is shown in Table 1. The parameter ranges for the STDP time windows were constrained by experimental data (Caporale and Dan, 2008) while the remaining parameter ranges were chosen to produce SNNs with biologically realistic firing rates.

**Table 1:** **Range of allowable values for parameters optimized by the automated parameter tuning framework**.

**Parameters**	**Range**
Max. Poiss. Rate	10–40 Hz
Buff → Exc Wts	4.0e-3–1.6e-2
Exc → Inh Wts	0.1–1.0
Inh → Exc Wts	0.1–0.5
R_target_ Exc	10–30 Hz
R_target_ Inh	40–100 Hz
A_+_ Exc	9.6e-6–4.8e-5
A_−_ Exc	9.6e-6–4.8e-5
τ_+_ Exc	10–60 ms
τ_−_ Exc	5–100 ms
A_+_ Inh	9.6e-6–4.8e-5
A_−_ Inh	9.6e-6–4.8e-5
τ_+_ Inh	10–60 ms
τ_−_ Inh	5–100 ms

Weight ranges and STDP *A*_+_ and *A*_−_ parameters are dimensionless and their relative magnitudes are important for creating a functional SNN.

The multi-component objective function was constructed by requiring output neurons to have desirable traits in neuronal response activity, namely, decorrelation, sparseness, and a description of the stimulus that employs the entire response range. The total fitness function to be maximized, fitness_total_, is described by Equation (1) and required each fitness component in the denominator to be minimized. Fitness values were normalized by the highest fitness value and ranged from 0 to 1. The fitness function consisted of three fitness components, fitness_decorr_, fitness_Gauss_, fitness_maxRate_, and a scaling factor *K* which had a value of 4.4 in all simulations discussed here.

(1) $${\text{fitness}}_{\text{total}}=\frac{1}{{\text{fitness}}_{\text{decorr}}+{\text{fitness}}_{\text{Gauss}}+K_{\text{scaling factor}}\cdot {\text{fitness}}_{\text{maxRate}}}$$

Here fitness_decorr_, described in Equation (2), was minimized if each output neuron responded uniquely and preferentially to a grating orientation, causing the average firing rates of each neuron to be decorrelated. The fitness component, fitness_Gauss_, was minimized when each Exc group neuron had an idealized Gaussian tuning curve response and is defined in Equation (4). The fitness component, fitness_maxRate_, was minimized when the maximum firing rate of the output neurons achieved a target firing rate, which helped neuronal activity remain stable and sparse, and is defined in Equation (6). A scaling term, K_scaling factor_ = 4.4, was used to correctly balance the maximum firing rate requirement against the decorrelation and Gaussian tuning curve curve requirements. Taken together, both fitness_maxRate_ and fitness_Gauss_ result in the assignment of high fitness values to neurons that have a stimulus response that utilizes the entire neuronal response range from approximately 0 to 60 Hz, which is an important aspect of neuronal activity.

The fitness_decorr_ component of the fitness function enforced decorrelation in the Exc group neuronal firing rates so that each neuron responded maximally to a different stimulus presentation angle. Equation (2) ensured the angles of maximum response θ^*i*^_max_ for each neuron, *i*, were as far from one another as possible by minimizing the difference between the two closest maximum angles (*D*^*i*^_min_) and the maximum possible value of *D*^*i*^_min_, called *D*_target_. *D*^*i*^_min_ is described in Equation (3) and *D*_target_ had a value of π/4.

(2) $${\text{fitness}}_{\text{decorr}}=\sum_{i=1}^{N=4}\left|D_{\min}^{i}-D_{\text{target}}\right|$$

(3) $$D_{\text{min}}^{i}=\min\left(\left|\theta_{\max}^{i}-\theta_{\max}^{j}\right|\right)\;\forall \;j\neq i$$

The next fitness component fitness_Gauss_ ensured that each Exc group neuron had a Gaussian tuning curve response similar to that found in V1 simple cells. The difference between the normalized firing rate *R^i^_j_* and a normalized Gaussian *G^i^_j_* was calculated for every presentation angle for each Exc group neuron and was summed over all angles and neurons. This is shown in Equation (4) while a description of the Gaussian is shown in Equation (5), where *r*^*i*^_max_ is the maximum firing rate for the *i*th neuron, θ^*i*^_max_ is the angle of maximum response of the *i*th neuron, θ_*j*_ is the *j*th stimulus angle, and σ was chosen to be 15π/180 to match experimental observations (Henry et al., 1974).

(4) $${\text{fitness}}_{\text{Gauss}}=\sum_{i=i}^{N=4}\sum_{j=1}^{M=40}\left|R_{\text{j}}^{i}-G_{\text{j}}^{i}\right|$$

(5) $$G_{j}^{i}=r_{\max}^{i}\exp\left[-\frac{1}{2}{\left(\frac{\theta_{j}-\theta_{\max}^{i}}{\sigma }\right)}^{2}\right]$$

The fitness_maxRate_ component, in combination with the Inh → Exc group connections, helped to enforce the requirement that the Exc group neurons had sparse firing rates by limiting the firing rate of each neuron to a maximum target firing rate *R*^max^_target_ of 60 Hz. The difference between the maximum firing rate *R*^*i*^_max_ of each Exc group neuron and the maximum target firing rate was calculated and summed over all Exc group neurons as shown in Equation (6).

(6) $${\text{fitness}}_{\text{maxRate}}=\sum_{i=1}^{N=4}\left|R_{\max}^{i}-R^{\max_{\text{target}}}\right|$$

Each fitness component had a fitness constraint imposed on it which caused the individual to be assigned a poor overall fitness if it fell outside a particular range of values. Recall that the fitness components are in the denominator of the total fitness equation making lower fitness component values more fit than higher fitness component values. The constraints are expressed as upper limits. Those individuals with fitness components larger than the upper limit were assigned poor overall fitness values by adding 240 to the denominator of Equation (1). The fitness component fitness_decorr_ had an upper limit constraint of 15, the fitness component fitness_Gauss_ had an upper limit of 1300, and the fitness component fitness_maxRate_ had an upper limit of 160.

### Network model

The input to the network consisted of a 32 × 32 grid of grayscale pixels, ranging from -1 to 1, which were connected to a pair of 32 × 32 Poisson spiking neuron groups with one-to-one topology to model the On/Off receptive fields found in the LGN. One Poisson spiking neuron group, the OnPoiss group, had linear spiking responses corresponding to Equation (7) while the OffPoiss group had responses corresponding to Equation (8). Here, *r*_*i*,On_ (*r*_*i*,Off_) represent the firing rate of neuron *i*, of the On(Off)Poiss group in response to the value of the input *p*, pixel *i*. The rates had maximum values of 1 and were scaled with the *Max. Poiss. Rate* parameter. Each On(Off)Poiss group had fixed excitatory synapses with one-to-one connectivity to a pair of 32 × 32 spiking neuron groups consisting of regular spiking (RS) Izhikevich neurons (Izhikevich et al., 2004), called the On(Off)Buffer groups. The On(Off)Buffer group neurons have a refractory period and were included to produce more realistic spike dynamics in response to the stimulus input. The On(Off) Buffer groups were included because Poisson spiking neurons with a built-in delay period were not part of the standard NVIDIA CUDA Random Number Generation (cuRAND) library and were therefore, more difficult to generate. On(Off)Buffer neurons had plastic excitatory synapses with all-to-all connectivity to an output group of RS neurons called the Exc group. Finally, to induce competition and encourage sparse firing, the Exc group made plastic excitatory all-to-all connections to a fast-spiking (FS) inhibitory neuron group (Izhikevich et al., 2004), which made fixed inhibitory all-to-all connections back to the Exc group.

(7) $$r_{i,\text{On}}\left(p_{i}\right)\;=\{\begin{array}{ll} p_{i}, & p_{i}>0\\ 0, & p_{i}\leq 0 \end{array}$$

(8) $$r_{i,\text{Off}}\left(p\right)\;=\{\begin{array}{ll} 0, & p_{i}>0\\ \left|p_{i}\right|, & p_{i}\leq 0 \end{array}\;$$

The mathematical description of the Poisson spiking neurons used in the simulation is shown in Equation (9).

(9) $$t_{i+1}=t_{i}-\ln\left(x_{i}\right)/r$$

The spike times were generated iteratively by generating interspike intervals (ISIs) from an exponential distribution (Dayan and Abbott, 2001). Here *t*_*i*_ is the spike time of the current spike, *t*_*i* + 1_ is the spike time of the next spike, *r* is the average firing rate, and *x*_*i*_ is the current random number (uniformly distributed between 0 and 1) used to generate the next spike time.

The spiking neurons used in the simulation were Izhikevich-type neurons and were chosen because they are computationally efficient and able to produce neural dynamics with a high degree of accuracy (Izhikevich, 2003). All excitatory neurons were modeled as RS neurons while all inhibitory neurons were modeled as FS neurons. The dynamics of Izhikevich neurons are shown in Equations (10, 11) and consist of a 2D system of ordinary differential equations.

(10) $$\frac{d\upsilon }{dt}=0.04\upsilon^{2}+5\upsilon +140-u+I\;$$

(11) $$\frac{du}{dt}=a(b\upsilon -u)$$

Here, υ is the membrane potential of the neuron and *u* is the recovery variable. The neuron dynamics for spiking are as follows:

If υ ≥ 30 mv, then $\{\begin{array}{l} \upsilon \leftarrow c\\ u\leftarrow u+d \end{array}$. The variables *a*, *b*, *c*, and d are specific to the type of Izhikevich neuron being modeled. For RS neurons, *a* = 0.02, *b* = 0.2, *c* = −65.0, and *d* = 8.0. For FS neurons, *a* = 0.1, *b* = 0.2, *c* = −65.0, and *d* = 2.0. The synaptic input for the spiking neurons consisted of excitatory NMDA and AMPA currents and inhibitory GABA_A_ and GABA_B_ currents (Izhikevich and Edelman, 2008) and has the form shown in Equation (12). Each conductance has the general form of *g*(υ − υ_0_) where *g* is the conductance, υ is the membrane potential, and υ_0_ is the reversal potential.

(12) $$\begin{array}{l} I=g_{\text{NMDA}}\frac{{\left[\frac{\upsilon +80}{60}\right]}^{2}}{1+{\left[\frac{\upsilon +80}{60}\right]}^{2}}(\upsilon -0)+g_{\text{AMPA}}\left(\upsilon -0\right)\\ \;+g_{{\text{GABA}}_{\text{A}}}\left(\upsilon +70\right)+g_{{\text{GABA}}_{\text{B}}}\left(\upsilon +90\right) \end{array}$$

The conductances obey the first order dynamics shown in Equation (13).

(13) $$\frac{dg_{i}}{dt}=-{\frac{g_{i}}{\tau }}_{i}$$

Here *i* denotes a particular conductance (NMDA, AMPA, GABA_A_, or GABA_B_) and τ denotes the decay constant for the conductance. The decay constants are τ_NMDA_ = 100 ms, τ_AMPA_ = 5 ms, τ_GABA__A_ = 6 ms, and τ_GABA__B_ = 150 ms.

All plastic connections used a standard nearest-neighbor STDP implementation (Izhikevich and Desai, 2003) but distinct STDP rules were used for STDP occurring between excitatory-to-excitatory (E → E) neurons and STDP occurring between excitatory-to-inhibitory (E → I) neurons. Excitatory-to-excitatory plastic connections had traditional STDP curves as detailed in (Bi and Poo, 1998) while excitatory-to-inhibitory plastic connections used STDP curves where potentiation occurred for pre-after-post pairings and depression occurred for pre-before-post pairings as found in experiments (Bell et al., 1997). A model for homeostatic synaptic scaling (Carlson et al., 2013) was also included to prevent runaway synaptic dynamics that often arise in STDP learning rules.

The STDP update rule used in our simulations is shown in Equation (14).

(14) $$\frac{dw_{i,j}}{dt}=\delta +\beta \left({\text{LTP}}_{i,j}+{\text{LTD}}_{i,j}\right)$$

The synaptic weight from presynaptic neuron *i* to postsynaptic neuron *j* is indicated by the variable *w*_*i,j*_. Additionally, δ is a bias often set to zero or a positive number to push the network toward positive weight increases for low synaptic input, while β is the learning rate. The weight changes were updated every time step (1 ms) but the weights themselves are modified once every 1 s.

To model homeostatic synaptic plasticity the STDP update rule was modified as shown in Equation (15) where α = 0.1 and β = 1.0.

(15) $$\frac{dw_{i,j}}{dt}=\left[\alpha \cdot w_{i,j}\left(1-\frac{\bar{R}}{R_{\text{target}}}\right)+\beta \left({\text{LTP}}_{i,j}+{\text{LTD}}_{i,j}\right)\right]\cdot \text{K}$$

Here, α is the homeostatic scaling factor while *R* and *R*_target_ are the average and target firing rates, respectively, for the postsynaptic neuron, *j*. A stability term denoted by K, damps oscillations in the weight updates and speeds up learning. K is defined as:

(16) $$\text{K}=\frac{\bar{R}}{T\cdot \left(1+\left|1-\bar{R}/R_{\text{target}}\right|\cdot \gamma \right)}$$

In Equation (16), γ is a tuning factor and *T* is the time scale over which the firing rate of the postsynaptic neuron is averaged. Here γ = 50 and *T* = 10 s.

### Simulation details

The SORF formation and performance analysis simulations were developed and implemented on a publically available neural simulator (Nageswaran et al., 2009b; Richert et al., 2011) and the forward Euler method (Giordano and Nakanishi, 2006) was used to integrate the difference equations with a step size of 1 ms for plasticity equations and 0.5 ms for neuronal activity equations. The CPU version of CARLsim was run on a system with an Intel Core i7 2.67 GHz quad-core processor with 6 GB of memory. The GPU version of CARLsim was run on a NVIDIA Tesla GPU M2090 card, with 6 GB of total memory and 512 cores. The GPU was capable of 665 GFLOPS of double precision, 1.33 TFLOPs of single precision, and had a memory bandwidth of 117 GB/s. The GPU was in a 12-core CPU cluster with 24 GB of memory and 4 GPU cards. Simulations executed on the CPU were single-threaded, while simulations executed on the GPU were parallelized, but only on a single GPU.

## Results

**Figure 4:**
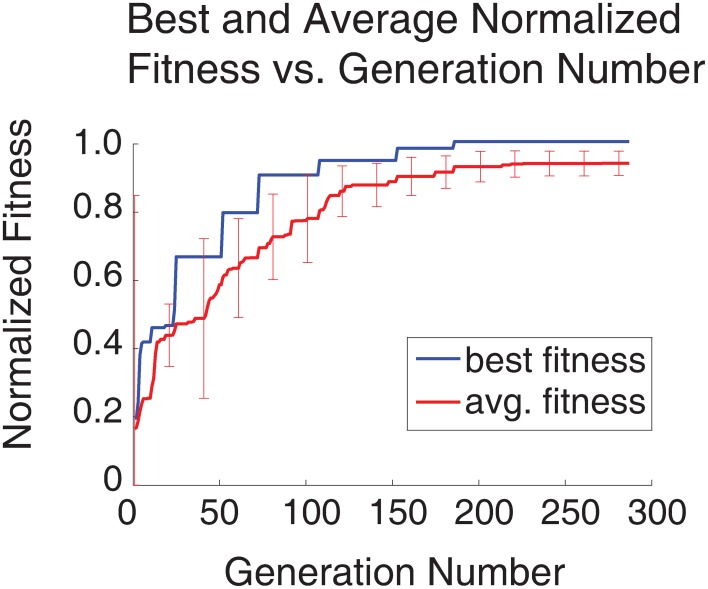
**Plot of best and average fitness vs. generation number for entire simulation run (287 generations, 4104 neuron SNNs, 10 parallel configurations)**. All values were normalized to the best fitness value. The error bars denote the standard deviation for the average fitness at intervals of once per 20 generations. Initially the standard deviation of the average fitness is large as the EA explores the parameter space, but over time, the standard deviation decreases as the EA finds better solutions.

An SNN capable of SORF formation and V1 simple cell like responses to counterphase grating stimuli presentation was constructed using the automated parameter tuning framework described above. Using a configuration with 10 SNNs running simultaneously on the GPU, each having 4104 neurons, the automated parameter tuning framework took 127.2 h to complete 287 generations of the EA and used a stopping criterion that halted the EA after the completion of 100 generations without a change in the fitness of the best individual or after the completion of 500 generations. The average and best fitness values for every generation are shown in red and blue, respectively, in Figure 4. The automated parameter tuning framework constructed 128 SNNs out of 2880 total SNNs (4.4%) that displayed SORF formation and V1 simple cell like responses and produced the first of these SNNs at generation 52. Table 2 shows the fitness values of the 10 initial SNNs and the fitness values after 287 generations. Shaded table entries denote SNNs that produced SORFs and V1 simple cell-like tuning curves for *all* four Exc group neurons while unshaded table entries indicate SNNs that failed to produce these neural phenomena. All SNNs from the initial population had very low fitness, produced no orientation selectivity, and had no SORF formation. All SNNs from the final population except for the last individual (fitness = 0.9040) had high fitness values, produced V1 simple cell-like tuning curve responses, and produced SORFs. The last individual in Table 2, had a high fitness, but only produced V1 simple-cell like tuning curve responses and SORFs in three of the four Exc group neurons.

**Table 2:**
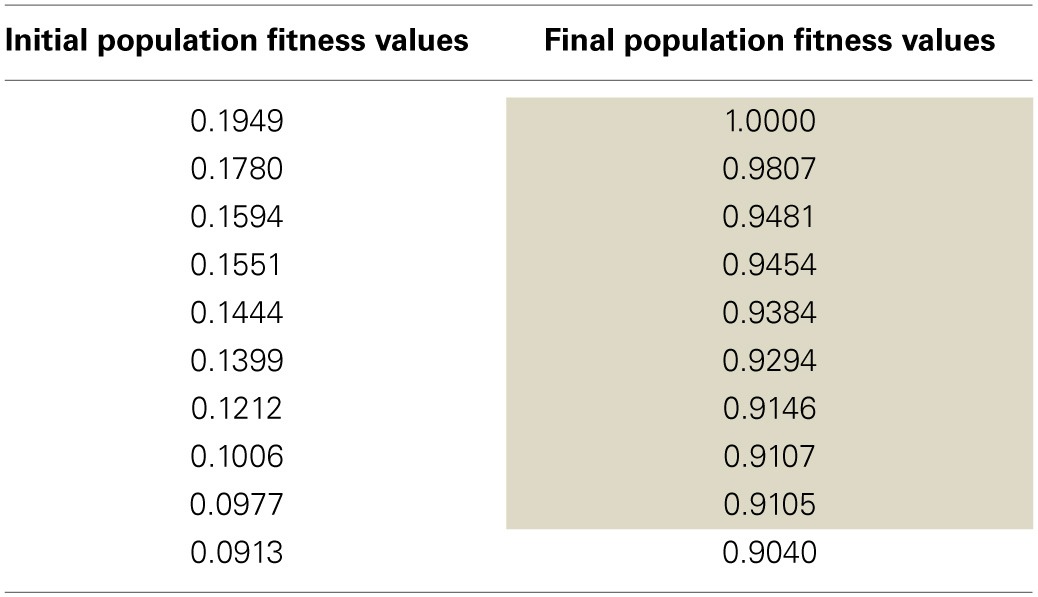
**Sorted fitness values (higher is better) for the initial and final SNN populations**.

Entries with a shaded background denote SNNs with V1 simple cell responses and SORF formation for every Exc group neuron (4).

### Evolving SNNs with V1 simple cell responses and SORF formation

**Figure 5:**
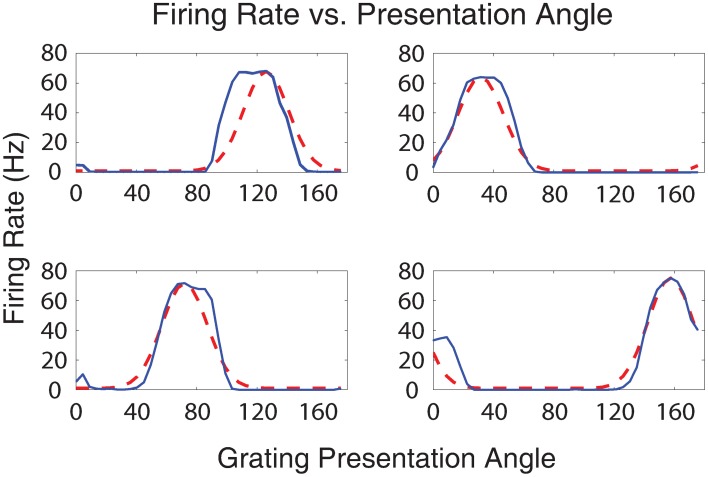
**Plot of the firing rate response of Exc group neurons vs. grating presentation orientation angle**. The blue lines indicate the firing rate of a neuron in the simulation while the dotted red lines indicate idealized Gaussian tuning curves. Together, the four excitatory neurons cover the stimulus space of all the possible presentation angles.

**Figure 6:**
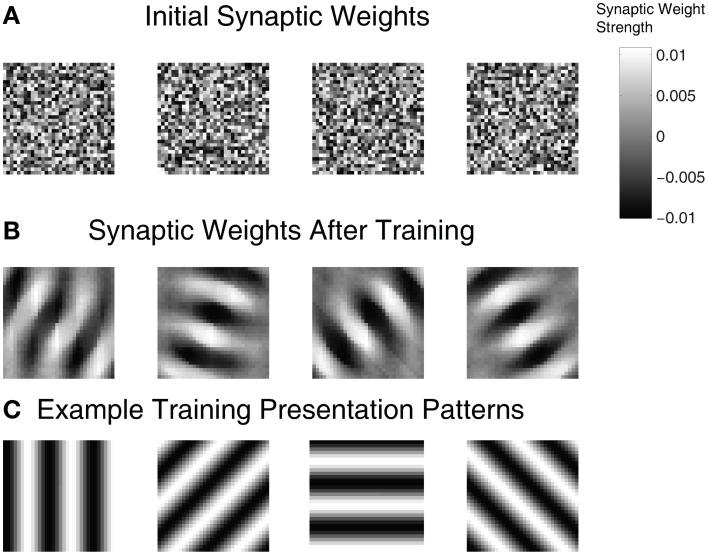
**Synaptic weights for the On(Off)Buffer → Exc connections of a high fitness SNN individual. (A)** Initial weight values before training. **(B)** After training for approximately 100 simulated minutes with STDP and homeostasis, the synaptic weight patterns resemble Gabor filters. **(C)** Four example orientation grating patterns are shown.

**Figure 7:**
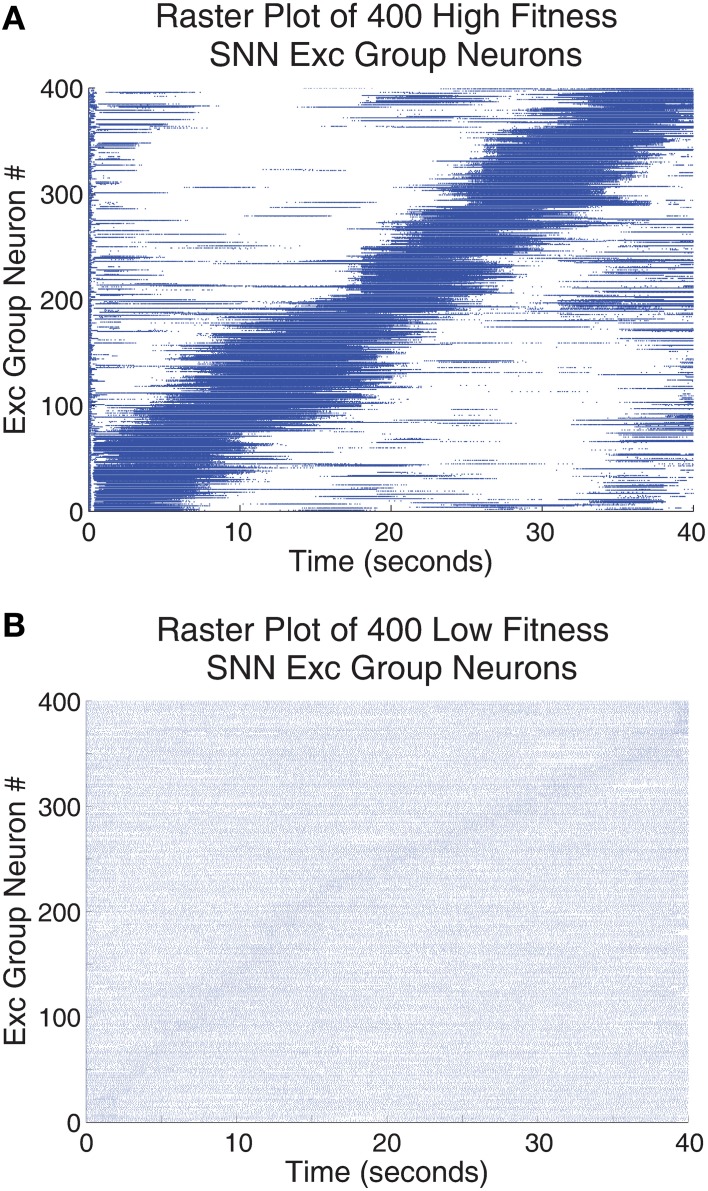
**The responses of the Exc group neurons (identified by their neuron id on the y-axis) were tested for all 40 grating orientations**. One orientation was presented per second and the test ran for 40 s (x-axis). **(A)** Neuronal spike responses of 400 neurons trained with the highest fitness SNN parameters found using the parameter tuning framework. **(B)** Neuronal spike responses of 400 neurons trained using a single set of low fitness parameters. The neurons were arranged such that those neurons responding to similar orientations were grouped together for both **(A,B)**. This accounts for the strong diagonal pattern found in **(A)** and the very faint diagonal pattern found in **(B)**. Neuronal spike responses in **(A)** are sparse in that relatively few neurons code for one orientation while neuronal spike responses in **(B)** are not sparse. Additional, many of the neuronal spike responses in part **(A)** employ a wide range of firing rates to describe a subset of the orientation stimulus space while spike responses in **(B)** have similar firing responses across all angles in all cases.

A single set of parameter values from the highest fitness individual (row 1, column 2 in Table 2) was used to generate Figures 5–7A, 10, these parameter values can be found in Supplementary 4 of the supplementary materials. Figure 5 shows the firing rates of four output neurons from the Exc group in response to all 40 input stimulus grating orientations. Each plot represents the firing rate of an individual Exc group neuron, denoted by a blue line, along with an idealized Gaussian tuning curve similar to those found in simple cell responses in visual cortical area V1 of the visual cortex (Henry et al., 1974), denoted by a dashed red line. The firing rate responses from the Exc group neurons qualitatively match the idealized V1 simple cell Gaussian tuning curves. The maximum firing rate responses of Exc group neurons were constrained by the sparsity requirement of the fitness function and peaked at an average value of 67 Hz. The firing rate responses were also decorrelated, another requirement of the fitness function, which lead to different preferred orientations for each of the Exc group neurons.

To examine the ability of the automated parameter tuning framework to construct SNNs capable of SORF formation, the synaptic weights between the On(Off)Buffer groups and the Exc group were visualized in Figure 6 for the highest fitness SNN. Each plot is a composite of the connections between the On(Off)Buffer group and a single Exc group neuron, where light regions represent strong synaptic connections and dark regions represent weak synaptic connections. Figure 6A shows the initial randomized synaptic weights while Figure 6B shows the final synaptic weights after 100 min of simulation time during the training period. The synaptic connections between the On(Off)Buffer neurons and the Exc neurons in Figure 6B formed receptive fields that resembled Gabor filters, which have been used extensively to model V1 simple cell responses (Jones and Palmer, 1987). Figure 6C shows four example counterphase sinusoidal grating orientations used as visual input into the SNN.

Figure 7 shows a raster plot of 400 Exc group neurons from 100 SNNs that were trained using the highest fitness parameter values taken from row 2, column 1 of Table 2 and shown in Figure 7A compared with a set of very low fitness parameters, fitness = 0.0978, shown in Figure 7B. Neurons that have similar preferred orientation angles have been placed close to one another. The high fitness neurons in Figure 7A have responses that are sparse (only a small subset of the neurons respond to any particular stimulus angle) and orthogonal (different neurons respond to different stimulus orientations) while neurons in Figure 7B do not have these properties. Although each high fitness neuron responds to a small subset of stimulus orientations, taken together the high fitness neurons have responses that cover all the possible stimulus orientations while low fitness neurons do not have responses that carry meaningful information in this respect.

**Figure 8:**
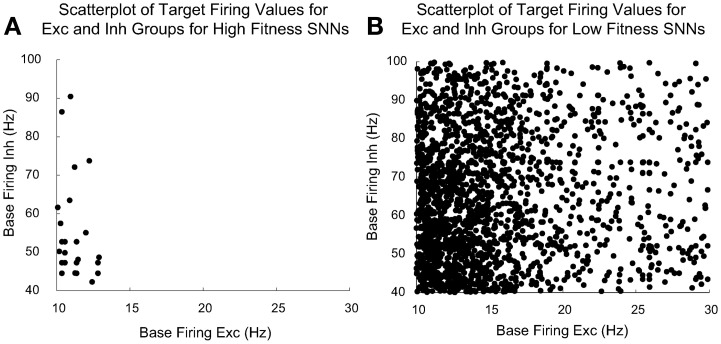
**Plot of the target homeostatic firing rate parameters for Exc group and Inh group for high fitness SNNs shown in (A) and low fitness SNNs shown in (B)**. The Exc group homeostatic target firing rate is significantly more constrained (between the ranges of 10–14 Hz) for the high fitness SNNs as opposed to the corresponding parameters for the low fitness SNNs. There were 128 high fitness SNNs and 2752 low fitness SNNs out of a total of 2880 individuals. EAs allow parent individuals to pass high value parameter values directly to their offspring, because of this, there are many offspring with identical high fitness values. This explains why there are not 128 distinct points distinguishable in **(A)**.

Figure 8 compares the evolved parameters of “high fitness” SNNs with “low fitness” SNNs. We judged an SNN to be high fitness if its three fitness component values met the following cutoffs: fitness_decorr_ had a cutoff value of 15, fitness_Gauss_ had a cutoff value of 950, and fitness_maxRate_ had a cutoff value of 50. We found these cutoffs produced SNNs with SORFs in the receptive fields of at least 3 out of 4 of the Exc group neurons. There were 128 high fitness SNNs and 2752 low fitness SNNs out of the 2880 total SNNs constructed and tested by the parameter tuning framework.

Figure 8 shows a comparison between homeostatic target firing rate parameters for Exc and Inh groups for high fitness SNNs (shown in Figure 8A) found using the parameter tuning framework along with the remaining low fitness parameter values (shown in Figure 8B). Each point represents a target Exc and Inh firing rate pair for a given SNN. The homeostatic target firing rate parameter for Exc groups in high fitness SNNs is clustered around a relatively small region (10–14 Hz) when compared to the total allowed target firing rate ranges of the Exc and Inh groups which are 10–30 and 40–100 Hz, respectively. The low fitness SNNs have Exc and Inh groups with target firing rates that have a much wider range of values. It is interesting that successful SNNs cluster around a low Exc group homeostatic firing rate (10–14 Hz). This may be due to the interplay between STDP time windows or the maximum input Poisson firing rate. In high fitness SNNs, Inh groups with higher homeostatic target firing rates are rare, but the distribution of firing rates is broader.

**Figure 9:**
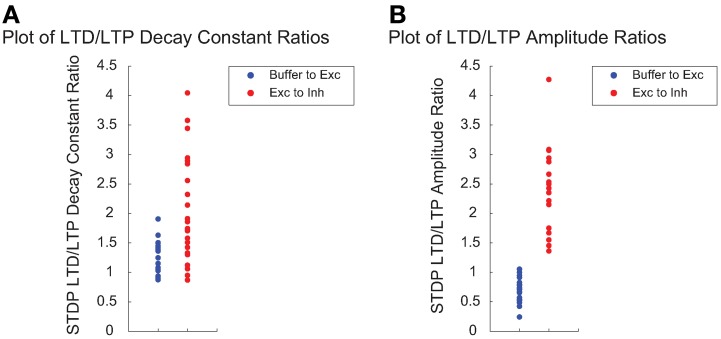
**The time windows in which STDP occurs are often modeled as decaying exponentials and each of the LTP and LTD windows can be characterized by single decay constant**. The degree to which the weight is increased during LTP or decreased during LTD is often called the LTP/LTD amplitude or magnitude. **(A)** Ratio of the STDP LTD/LTP decay constant for the Buffer to Exc group connections (blue) and the Exc to Inh group connections (red) for high fitness SNNs. **(B)** The ratio of the STDP LTD/LTP amplitude for the Buffer to Exc group connections (blue) and the Exc to Inh group connections (blue) for high fitness SNNs.

We next examined the relationship between STDP plasticity parameters among high fitness SNNs individuals exclusively. Figure 9A shows the LTD/LTP decay constant ratios, which dictate the size of the LTP and LTD time windows, for Buffer to Exc group connections and Exc to Inh group connections. Figure 9B shows a comparison between LTD/LTP amplitude ratios for Buffer to Exc group connections and Exc to Inh group connections. The overall parameter ranges can be found in Table 1. The Buffer to Exc decay constant ratio in Figure 9A is within close range of experimental observations by (Bi and Poo, 1998), that show the LTD decay constant as being roughly twice as large at the LTP decay constant. The Exc to Inh LTD/LTP decay constant ratio in Figure 9A has a broader distribution of values that ranged from approximately 1 to 4. These values also fall within the range of experimental measurements of the LTD/LTP decay constant ratio of approximately one (Bell et al., 1997). High fitness SNNs in Figure 9B show a narrow distribution of LTD/LTP amplitude ratios that favor an LTD/LTP ratio less than one for Buffer to Exc group connections while Exc to Inh group connections show significantly broader LTD/LTP amplitude ratios with values ranging from approximately 1 to 4.

### Stability analysis

To ensure that the solutions found by the automated tuning framework were stable, the parameter set from the highest fitness SNN was used to train and test an SNN for an additional 100 trials, allowing the SORFs to be learned through STDP and tested as described in the previous section. That is, a single set of parameters was tested to ensure that the ability of a naïve SNN to form SORFs was repeatable and independent of stimulus presentation order. Thus, the order of stimulus presentations was randomized between trials and each trial consisted of training and testing phases. Parameter values were deemed stable if the SNNs consistently produced V1 simple cell-like responses and SORFs for the majority of the trials. A robustness analysis on the effect of small perturbations on the functional behavior of the SNNs was not performed. To further analyze the stability of the parameter values, firing rate responses from the Exc group over all 100 trials were used to decode eight test angles presented to the trained SNNs with a widely used population decoding algorithm (Dayan and Abbott, 2001). At each presentation of the eight orientation test angles, the neuronal firing rate and its preferred stimulus orientation (i.e., the orientation for which the neuron fired maximally) were used to create a population vector for all the Exc neurons from the 100 trials (4 Exc neurons per trial × 100 trials = 400 neurons in total). The neuronal population vectors were averaged and the resultant vector was compared to the stimulus orientation angle.

**Figure 10:**
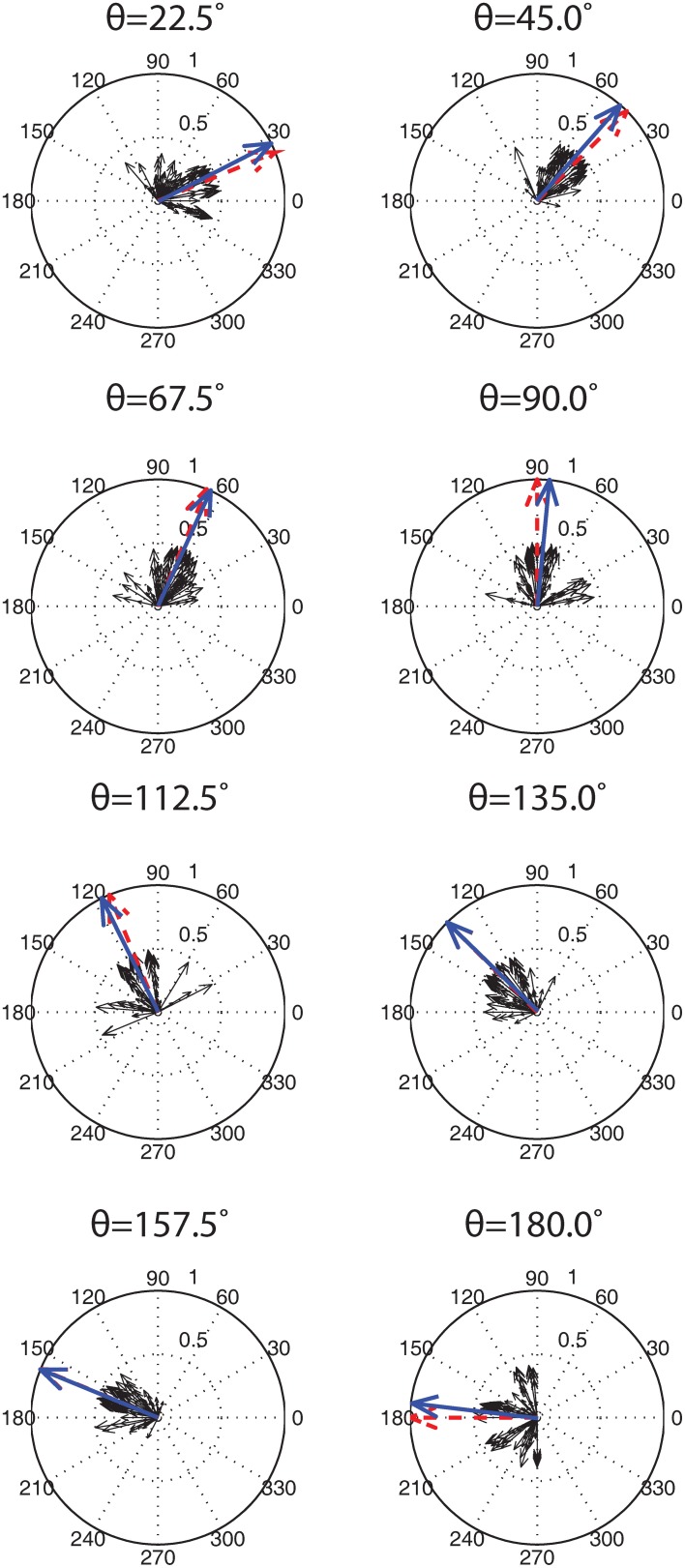
**Population decoding of eight test presentation angles**. The test presentation angle θ, is shown above each population decoding figure. 100 simulation runs, each with identical parameter values but different training presentation orders, were conducted and the firing rates of the Exc group neurons were recorded. The individual responses of each of the 400 neurons (4 Exc neurons × 100 runs) are shown with solid black arrows. These individuals were summed to give a population vector (shown with a blue arrow) that was compared to the correct presentation angle (shown with a red arrow). Both the population vectors and correct presentation angle vectors were normalized while the component vectors were scaled down by a factor of 2 for display purposes (see text for details).

The results of the 100 training and testing trials for the identical set of parameters were as follows. 76% of the trials had SNNs with tuning curves that qualitatively matched V1 simple cell responses and produced Gabor filter-like SORFs. The remaining 24% of the trials had SNNs with three Exc group neurons that produced good behavior and a single Exc group neuron with a bimodal tuning curve and a SORF that resembled two overlapping Gabor filters at different angles. A population code from the firing rate responses of the 400 Exc group neurons was used to decode the orientation of the presented test stimuli. Figure 10 shows the population decoding results for eight presented test angles. The smaller black arrows are neuronal responses from the 400 neurons which sum to the population vector, shown with a blue arrow. The lengths of the individual neural response vectors (black arrows) were normalized by dividing the mean firing rate by 2. The length of the population vector (blue arrow) was normalized by dividing the sum of the individual responses by the magnitude of the vector. The population vector was very close to the presented test stimulus orientation for every case with a mean error of 3.4° and a standard deviation of 2.3°.

### Performance analysis

To test the computational performance of the automated parameter tuning framework, three different sized SNNs were run using either a serial CPU implementation or a parallel GPU implementation of CARLsim. Each SNN had identical topology except for the size of the On(Off)Poiss and On(Off)Buffer groups which were either 16 × 16, 24 × 24, or 32 × 32 giving rise to networks with 1032, 2312, and 4104 neurons, respectively. The number of configurations executed in parallel on the GPU was varied from 5 to 30 for all network sizes and execution times were recorded.

**Figure 11:**
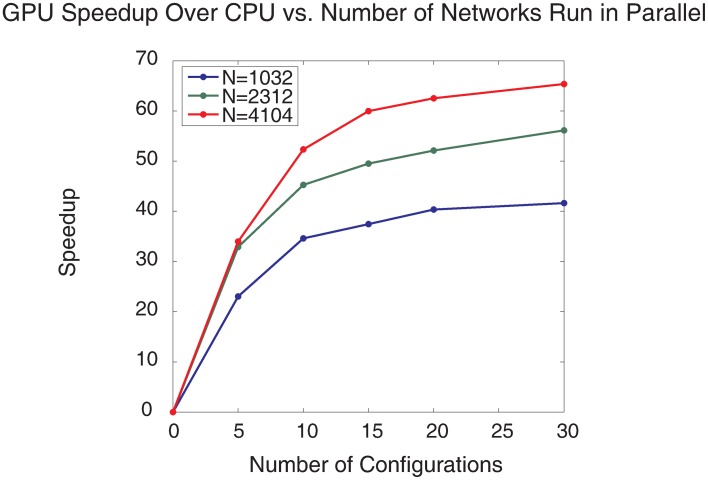
**Plot of GPU speedup over CPU vs. number of SNNs run in parallel for different sized SNNs and different numbers of SNNs run in parallel**. Three different SNN sizes were used, the blue line denotes SNNs with 1032 neurons, the green line denotes SNNs with 2312 neurons, and the red line denotes SNNs with 4104 neurons.

The parallelized GPU SNN implementation showed impressive speedups over the CPU SNN implementation (Figure 11). The largest speedup (65×) was found when 30 SNN configurations, each with 4104 neurons, were run in parallel, which took 21.1 min to complete a single generation, whereas 10 SNN configurations with 4104 neurons required 26.4 min to complete a single generation. In contrast, the CPU took 23.5 h for a single generation. It would be interesting to compare the GPU performance with a multi-threaded CPU simulation and there may be gains in such an approach. However, in our experience SNNs on such systems do not optimize or scale as well as GPUs. Because the calculation of SNN neuronal and synaptic states can be cast as single instruction multiple data (SIMD), parallel computation of SNNs is more suited to GPUs having thousands of simple cores, rather than multithreaded CPUs having many less, but more powerful cores.

As the number of concurrent SNN configurations grows, the speedup increases slowly and nearly plateaus for 30 parallel SNN configurations. These speedup plateaus are mostly likely due to the limitations of the GPU core number and clock-frequency, and not the GPU global memory size as 99% of the GPU was utilized but less than 20% of the GPU memory was utilized for the largest simulation configurations. It should be noted that although the single SNN configuration was moderately sized, all 30 configurations together comprised a large-scale network (i.e., 123,120 total neurons) that was running simultaneously. This parameter tuning approach can be scaled to tune larger SNNs by running fewer configurations in parallel or by spreading the computation and memory usage across multiple GPUs with an MPI/CUDA implementation.

## Discussion

With the growing interest in large-scale neuromorphic applications using spiking neural networks, the challenge of tuning the vast number of open parameters is becoming increasingly important. We introduced an automated parameter tuning framework that can quickly and efficiently tune SNNs by utilizing inexpensive, off-the-shelf GPU computing technology as a substitute for more expensive alternatives such as supercomputing clusters. The automated parameter tuning framework consists solely of freely available open source software. As a proof of concept, the framework was used to tune 14 neural parameters in an SNN ranging from 1032 to 4104-neurons. The tuned SNNs evolved STDP and homeostasis parameters that learned to produce V1 simple cell-like tuning curve responses and SORFs. We observed speedups of 65× using the GPU for parallelization over a CPU. Additionally, the solutions found by the automated parameter tuning framework were shown to be stable.

There are a few research groups that have designed software frameworks capable of tuning large-scale SNNs. Eliasmith et al. (2012) constructed a 2.5 million neuron simulation that demonstrated eight diverse behavioral tasks by taking a control theoretic approach called the Neural Engineering Framework (NEF) to tune very large-scale models. The NEF is implemented in a neural simulator called Nengo and can specify the connection weights between two neuronal populations given the input population, the output population, and the desired computation to be performed on those representations. Our parameter tuning framework takes a different approach, allowing the user to tune not only individual synaptic weights but also parameters related to plasticity rules, connection topology, and other biologically relevant parameters. Our framework does not require the user to specify the desired computations between two neuronal populations but rather leaves it to the user to specify the exact fitness function. The popular SNN simulator, Brian (Goodman and Brette, 2009), also has support for parameter tuning in the form of a parallelized CPU/GPU tuning toolkit. Their toolkit has been used to match individual neuron models to electrophysiological data and also to reduce complex biophysical models to simple phenomenological ones (Rossant et al., 2011). Our tuning framework is focused more on tuning the overall SNN behavior as opposed to tuning a spiking model neuron that captures electrophysiological data.

SNNs constructed and tuned with our framework could be converted to run on any neuromorphic device that incorporates the AER format for spike events and supports basic connection topologies. This is the case for many neuromorphic hardware devices (Furber et al., 2012; Cruz-Albrecht et al., 2013; Esser et al., 2013; Pfeil et al., 2013). Although the framework presented here was run on the CARLsim simulator, which utilizes the Izhikevich neuron and STDP, the automated tuning framework presented here could readily be extended to support any spiking model, such as the leaky integrate-and-fire neuron or the adaptive exponential integrate-and-fire neuron (Brette and Gerstner, 2005).

SNNs with thousands of neurons, multiple plasticity rules, homeostatic mechanisms, and feedback connections, similar to the SNN presented here, are notoriously difficult to construct and tune. The automated parameter tuning framework presented here can currently be applied to much larger SNNs (on the scale of 10^6^ neurons) with more complex network topologies but GPU memory constraints limit the tuning of larger SNNs. Currently, CARLsim SNN simulations are limited to approximately 500 K neurons and 100 M synapses on a single Tesla M2090 GPU, but a version that allows SNN simulations to run across multiple GPUs is in development and will increase the size of SNNs that can be tuned using this framework. The combination of a multi-GPU version of CARLsim and the implementation of more advanced evolutionary computation principles, such as multi-objective fitness functions and co-evolving populations, should allow the framework to be scalable and capable of tuning large-scale SNNs on the scale of millions of neurons. The highly efficient automated parameter tuning framework presented here can reduce the time researchers spend constructing and tuning large-scale SNNs and could prove to be a valuable contribution to both the neuromorphic engineering and computational neuroscience research communities.

### Conflict of interest statement

The authors declare that the research was conducted in the absence of any commercial or financial relationships that could be construed as a potential conflict of interest.
