# Supplementary material for: An efficient automated parameter tuning framework for spiking neural networks
Source: Front Neurosci. 2014 Feb 4;8:10. doi: 10.3389/fnins.2014.00010 (PMC3912986; doi:10.3389/fnins.2014.00010)
Supplement: Supplementary file 1 [file DataSheet1.DOCX]

**Supplementary Material**

**Supplementary 1. EO parameter file configuration for all tuning simulations**

Below is the EO parameter file used for all tuning simulations presented here. The green text denotes commented code. There is an additional section entitled ‘Genotype Initialization’ that was not included because it is ignored by our PTI which relies on explicit, user-defined parameter initializations. All parameter file options commented out are assigned default values and explained in the documentation of EO.

## Sample parameter file to implement the mu + lambda ES algorithm. ##
###### General ######
--help=0 # -h : Prints this message
--stopOnUnknownParam=1 # Stop if unkown param entered
--seed=1104133126 # -S : Random number seed

###### ES mutation ######
--Isotropic=1 # -i : Isotropic self-adaptive mutation
--Stdev=1 # -s : One self-adaptive stDev per variable
--Correl=0 # -c : Use correlated mutations

###### Evolution Engine ######
--popSize=10 # -P : Population Size
--selection=DetTour(2) # -S : Selection: DetTour, StochTour, Roulette, Ranking, Seq.
--nbOffspring=100% # -O : Nb of offspring (percentage or absolute)
--replacement=Plus # -R : Replacement: Comma, Plus, EPTour, SSGAWorst, SSGADet
--weakElitism=1 # -w : Old best parent replaces new worst offspring

###### Output ######
--useEval=1 # Use nb of eval. as counter (vs nb of gen.)
--useTime=1 # Display time (s) every generation
--printBestStat=0 # Print Best/avg/stdev every gen.
--printPop=0 # Print sorted pop. every gen.

###### Output - Disk ######
--resDir=EA-Data # Directory to store DISK outputs
--eraseDir=0 # erase files in dirName if any
--fileBestStat=1 # Output bes/avg/std to file

###### Output - Graphical ######
--plotBestStat=0 # Plot Best/avg Stat
--plotHisto=0 # Plot histogram of fitnesses

###### Persistence ######
# --Load= # -L : A save file to restart from
# --recomputeFitness=0 # -r : Recompute the fitness after re-loading the pop.?
# --saveFrequency=0 # Save every F generation (0=only final state, absent=never)
# --saveTimeInterval=0 # Save every T seconds (0 or absent = never)
# --status=t-eoESAll.status # Status file

###### Stopping criterion ######
--maxGen=500 # -G : Maximum number of generations () = none
--steadyGen=200 # -s : Number of generations with no improvement
--minGen=0 # -g : Minimum number of generations
--maxEval=0 # -E : Maximum number of evaluations (0 = none)
--targetFitness=400 # -T : Stop when fitness reaches
--CtrlC=0 # -C : Terminate current generation upon Ctrl C

###### Variation Operators ######
# --objectBounds=10[-inf,+inf] # -B : Bounds for variables
# --operator=SGA # -o : Description of the operator (SGA only now)
--pCross=0.4 # -C : Probability of Crossover
--pMut=0.4 # -M : Probability of Mutation
#--crossType=global # -C : Type of ES recombination (global or standard)
#--crossObj=discrete # -O : Recombination of object variables
#--crossStdev=intermediate # -S : Recombination of mutation strategy parameters
#--TauLoc=1 # -l : Local Tau (before normalization)

**Supplementary 2. Basic Automated Parameter Tuning Sample Program Outline**

Figure I describes the basic structure of a program that tunes SNNs by using the PTI API (Algorithm 1). Before Algorithm 1 is run, the PTI and CARLsim header files are first included in the main source file to provide the user with access to the appropriate function calls. Algorithm 1 begins with the construction of CARLsim SNNs and the reading of the configured EO parameter file (Steps 1 – 2). Information on how to build and run SNNs using CARLsim is described in (Richert et al., 2011). Next, a ParamTuning object is instantiated and the parameters to be optimized are created and registered (Steps 3 – 4). After all user-defined parameters have been created, the ParamTuning object sets the total number of parameters, updates the genome size, and selects the appropriate EA algorithm (Step 5). Two iteration statements follow: the outer loop iterates over every EA generation while the inner loop iterates over every SNN configuration to be run in parallel (Steps 6 and 7). Within the inner loop, PTI functions access EO parameter values and assign them to CARLsim SNN parameter values followed by the loop termination (Steps 8 – 10). Within the outer loop, SNNs with newly assigned parameter values are run in parallel (Step 11), the fitness is evaluated for each SNN in parallel (step 12), the fitness values are passed from CARLsim to EO (Step 13), the EA is run for the next generation (14), and the loop is exited (Step 15). Algorithm 1 ends when one of the termination conditions is met, resulting in the completion of the EA and the printing of EA population and individual data. Supplementary 2 of the supplementary materials includes a summary of the available PTI commands with a short description on how to use them


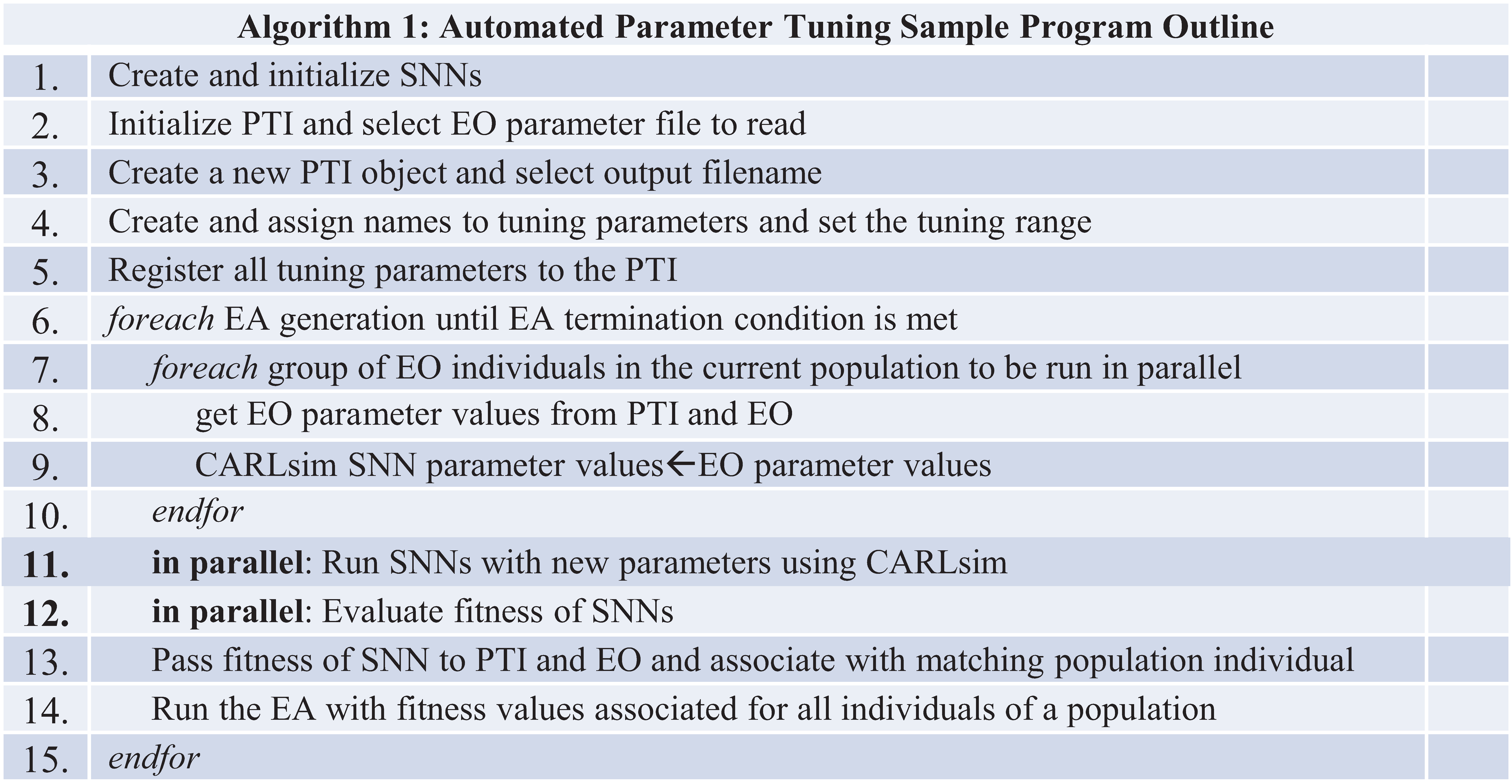


**Figure I**: Basic automated parameter tuning sample program outline. Steps 11 and 12 were run in parallel using a GPU accelerated version of the CARLsim SNN simulator.

**Supplementary 3. PTI-API activity summary**

**Table I**: A summary of the PTI-API. The table is organized into three categories, Activity, Example, and Comments. Lines labeled ‘Activity’ describe the task or step that needs to be completed by Algorithm 1. All blue shaded lines denote ‘Example’ lines where sample code is listed. Finally, lines labeled with ‘Comments’ briefly explain the meaning of the example code.

| PTI-API Activity Summary | | |
| --- | --- | --- |
| 1. | **Activity:** Include PTI header file |  |
|  | **Example:** #include "pti.h" |  |
|  | **Comments:** This is the only header file inclusion required to use the PTI in CARLsim. |  |
|  |  |  |
| 2. | **Activity:** Initialize PTI and select EO parameter file to read |  |
|  | **Example:** InitializeParamTuning("EO_Param_File.param"); |  |
|  | **Comments:** This command must always before all other PTI API function calls as low-level configurations are made during this function call. |  |
|  |  |  |
| 3. | **Activity:** Create a new PTI object and select output filename |  |
|  | **Example:** ParamTuning *p = new ParamTuning("EO_Output.txt"); |  |
|  | **Comments:** A pointer to a ParamTuning object p is created. EA statistics will be output to the file ‘EO_Output.txt’. |  |
|  |  |  |
| 4. | **Activity:** Create and assign names to tuning parameters and set the tuning range |  |
|  | **Example:** p->addParam("inhWt", 0.1, 0.5); |  |
|  | **Comments:** addParam adds tuning parameter named ‘inhWt’ and sets the tuning range to be 0.1—0.5. |  |
|  |  |  |
| 5. | **Activity:** Register all tuning parameters to the PTI |  |
|  | **Example:** p->updateParameters(); |  |
|  | **Comments:** This must be called before the evolutionary algorithm gets run as it makes all the tuning parameters known to EO. |  |
|  |  |  |
| 6. | **Activity:** Assign EO parameter values to CARLsim SNN parameter values |  |
|  | **Example:** inhWeight[configId] = -1.0*(p->getParam(IndiId,s1="inhWt")); |  |
|  | **Comments:** This assignment takes place inside the innermost loop of Algorithm 1. ‘inhWeight’ is a CARLsim SNN parameter array indexed by ‘configId’ which represents the specific ID of one of the SNNs to be run in parallel. The getParam function takes the EO individual ID and string identifier of the tuning parameter and returns the value of this tuning parameter. The variable ‘IndiId’ indexes the current population individuals. |  |
|  |  |  |
| 7. | **Activity:** Evaluate fitness of SNNs |  |
|  | **Example:** evaluateFitness(snn, fitness); |  |
|  | **Comments:** This function must be written by the user because fitness functions essentially define the optimization problem. Here we pass a spiking neural network object defined in CARLsim named ‘snn’ and a fitness array to the evaluateFitness function. The evaluateFitness function runs the SNNs and assigns their fitness values to the fitness array. |  |
|  |  |  |
| 8. | **Activity:** Pass fitness of SNN to EO and associate with matching population individual |  |
|  | **Example:** p->setFitness(fitness, IndiId, NUM_CONFIG); |  |
|  | **Comments:** The setFitness function associates the fitness array ‘fitness’ with the currently evaluated population individuals and SNNs. ‘IndiId’ is the index of the EA population of the current generation and ‘NUM_CONFIG’ is the total number of SNN configurations to be run in parallel. |  |
|  |  |  |
| 9. | **Activity:** Run the EA with fitness values associated for all individuals of a population |  |
|  | **Example:** bool continueIter = p->runEA(); |  |
|  | **Comments:** This function is run at the end of every EA generation loop (the outer loop in Algorithm 1). Because EO now has the fitness values of the individuals of the current population, it can be called to select, breed, recombine, and mutate parameters for the next generation. It returns a Boolean value that is true if no termination conditions have been met and the generation loop should continue and false if at least one termination condition has been met and the EA should be halted. |  |
|  |  |  |
| 10. | **Activity:** Output summary statistics of EA |  |
|  | **Example:** p->printSortedPopulation(); |  |
|  | **Comments:** This function outputs the population fitness sorted by fitness. |  |

**Supplementary 4. Parameter values from high and low fitness SNNs**

**Table II.** Parameter values from the highest fitness SNN (fitness = 1.0) and a low fitness SNN (fitness = 0.0978) found using the automated parameter tuning framework. These SNN parameter values are used whenever data from a single SNN are analyzed throughout the paper. $A_{-}$ Exc, $A_{-}$ Inh, and Inh🡪Exc Wts have negative values when used in CARLsim.

| **Parameters** | **High Fitness SNN Values** | **Low Fitness SNN Values** |
| --- | --- | --- |
| Max. Poiss. Rate | 25.9 Hz | 34.4 Hz |
| Buff🡪Exc Wts | 1.5e-2 | 1.3e-2 |
| Exc🡪Inh Wts | 5.3e-1 | 8.0e-1 |
| Inh🡪Exc Wts | 1.7e-1 | 4.6e-1 |
| R_target_ Exc | 10.3 Hz | 21.1 Hz |
| R_target_ Inh | 47.3 Hz | 75.7 Hz |
| A_+_ Exc | 3.5e-5 | 4.0e-5 |
| A_-_ Exc | 1.7e-5 | 2.5e-5 |
| τ_+_ Exc | 43.2 ms | 33.1 ms |
| τ_-_ Exc | 45.7 ms | 54.3 ms |
| A+ Inh | 1.4e-5 | 3.1e-5 |
| A- Inh | 4.3e-5 | 2.2e-5 |
| τ_+_ Inh | 11.2 ms | 46.7 ms |
| τ_-_ Inh | 19.0 ms | 73.3 ms |

**References**

Richert, M., Nageswaran, J. M., Dutt, N., and Krichmar, J. L. (2011). An efficient simulation environment for modeling large-scale cortical processing. *Front. Neuroinformatics* 5. doi: 10.3389/fninf.2011.00019..
